# Large-Scale Protein Interactions Prediction by Multiple Evidence Analysis Associated With an In-Silico Curation Strategy

**DOI:** 10.3389/fbinf.2021.731345

**Published:** 2021-09-06

**Authors:** Yasmmin Côrtes Martins, Artur Ziviani, Marisa Fabiana Nicolás, Ana Tereza Ribeiro de Vasconcelos

**Affiliations:** ^1^ Bioinformatics Laboratory, National Laboratory of Scientific Computing, Petrópolis, Brazil; ^2^ Data Extreme Lab (DEXL), National Laboratory of Scientific Computing, Petrópolis, Brazil

**Keywords:** protein-protein interaction, scientific workflow, PPI prediction, text mining, in-silico validation, large-scale prediction

## Abstract

Predicting the physical or functional associations through protein-protein interactions (PPIs) represents an integral approach for inferring novel protein functions and discovering new drug targets during repositioning analysis. Recent advances in high-throughput data generation and multi-omics techniques have enabled large-scale PPI predictions, thus promoting several computational methods based on different levels of biological evidence. However, integrating multiple results and strategies to optimize, extract interaction features automatically and scale up the entire PPI prediction process is still challenging. Most procedures do not offer an *in-silico* validation process to evaluate the predicted PPIs. In this context, this paper presents the PredPrIn scientific workflow that enables PPI prediction based on multiple lines of evidence, including the structure, sequence, and functional annotation categories, by combining boosting and stacking machine learning techniques. We also present a pipeline (PPIVPro) for the validation process based on cellular co-localization filtering and a focused search of PPI evidence on scientific publications. Thus, our combined approach provides means to extensive scale training or prediction of new PPIs and a strategy to evaluate the prediction quality. PredPrIn and PPIVPro are publicly available at https://github.com/YasCoMa/predprin and https://github.com/YasCoMa/ppi_validation_process.

## 1 Introduction

Proteins are complex macromolecules that play an essential role in the cellular machinery, perform functions in biological processes Safari-Alighiarloo et al. (2014), and regulate gene expression under certain conditions (Cooper, 2000). While many proteins may execute their function individually, other proteins either physically bind to or functionally associate with each other, thereby producing protein-protein interactions (PPIs) to perform their function correctly. Currently, *in-silico* bioinformatics approaches represent an efficient method of detecting PPIs on a large scale and facilitating the best candidate pairs’ prioritization for posterior experimental validation. PPI detection methods that combine multiple pieces of evidence such as evolution, functional characteristics, structural features, and sequence-based methods, have achieved better performance than other approaches that only use one or few pieces of evidence (Chang et al., 2016).

The most recent PPI detection methods are based on machine learning techniques (Kotlyar et al., 2015; Arango-Rodriguez et al., 2016; Hashemifar et al., 2018; Chen et al., 2019; Wang et al., 2019; Li and Ilie, 2020). A few methods (Kotlyar et a.,. 2015; Chen et al., 2019) also use various protein features to predict PPIs, combining functional annotations with network topology and others such as orthology and paralogy. Some related works typically use a non-automatic and costly feature extraction step to generate the prediction inputs (Hashemifar et al., 2018; Chen et al., 2019). Some recent results (Gonzalez-Lopez et al., 2018; Chen et al., 2019; Yang et al., 2020) offer automatic feature extraction but do not provide a mechanism to reuse the already calculated features to optimize subsequent experiments according to preliminary information. Some predictors offer large-scale prediction focusing only on optimizing training and evaluation predictors but their strategies have not considered the data distribution in independent processes in parallel (Chen et al., 2008; Pan et al., 2010; You et al., 2014; Zhang et al., 2014). Other methods (Papanikolaou et al., 2015) identify PPIs using text mining techniques, although most of these methods still present a high number of false-positive results. These works also start from a global search to discover any possible interaction from the texts, which may be time-consuming. Finally, few methods (Tan et al., 2004; Antony et al., 2008; Frech et al., 2009) analyze predicted PPIs to perform postprocessing validation and assist in their curation process.

We introduce a new PPI prediction method, PredPrIn (Prediction of Protein Interactions), a scientific workflow for end-to-end data management from preprocessing to PPIs classification. The main goal of PredPrIn is executing large-scale protein interaction prediction, acting in training/prediction modes. PredPrIn automatically extracts protein information to create a reusable and adaptable knowledge base, enhancing the speed of further experiments according to the diversity of proteins in the database. Our method combines four types of detection methods (based on the primary sequencee (Li and Ilie, 2017), the semantic similarity of Gene Ontology terms (Pekar and Staab, 2002), domain interactions and co-participation in pathways) as the base-level predictors of the stacked generalization scheme (Džeroski and Ženko (2004)) and uses a meta-level classifier based on the boosting technique (Schapire, 2013). Complementing the PredPrIn method, we also present a validation process based on cell location co-occurrence filtering and a focused search on individual PPIs’ relevant scientific publications. The text mining part was projected with context filtering to eliminate false-positive relations from other regulation events between proteins.

## 2 Materials and Methods

### 2.1 PredPrIn

**FIGURE 1:**
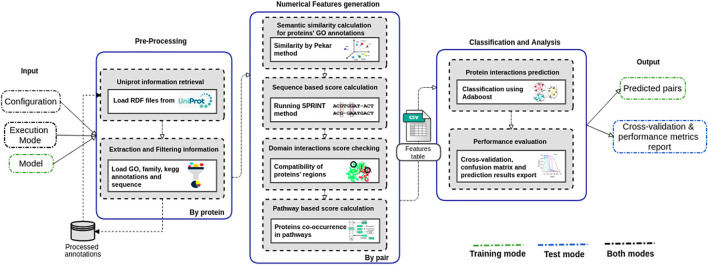
Steps of the PredPrIn architecture: (i) Preprocessing; (ii) Numerical feature generation, and (iii) Classification and analysis. The preprocessing step retrieves protein information from the UniProt database, and automatically parses and filters them to build a knowledge base of processed features. The numerical feature generation step uses this knowledge base to distribute the correct inputs for the execution of the four detection methods and then generates a matrix of scores organized according to these methods’ results. This matrix is used in the classification and analysis step to carry out the prediction by a classifier implementing the boosting technique. PredPrIn may run in either training or test mode. In both modes it is mandatory fulfilling the configuration file with the information of the datasets containing PPIs and the specification of mode type (train or test). In test mode, the additional input is a trained model previously computed and the workflow output is the positively predicted PPIs. No further input is required in training mode, and its outputs correspond to the trained model, the cross-validation and performance metrics report.

The PredPrIn (Figure 1) architecture is divided into three steps, namely 1) Preprocessing; 2) Numerical feature generation; and 3) Classification and analysis. Our method deals with protein data acquisition, feature computation according to diversified PPI detection methods, and result exportation of PPI classification.

#### 2.1.1 Preprocessing

We use the functional annotations retrieved from the UniProt^1^ database to create a preprocessed feature knowledge base to be reused for subsequent prediction experiments. PredPrIn stores RDF (resource description framework) files (Cyganiak et al., 2014) for each protein of the interaction pairs in the workflow input. The Extraction and Filtering information module automatically parses these files running SPARQL (Group, 2013) queries to obtain the properties related to functional annotations, amino acid sequence, and identification in the Pfam^2^ as well in the Kegg Orthology^3^ (KO) databases. The results of these queries are then filtered according to the required inputs of the detection methods, and the final list of features is stored in the knowledge base. We also added a controller to check whether updated information on the protein is already included in our knowledge base. Each time a new experiment is started, this controller checks the RDF files’ integrity hashes to compare local and UniProt versions.

#### 2.1.2 Numerical Features Generation

PredPrIn computes numerical features for classification using four types of detection methods that are based on the primary sequence of amino acids (SPRINT–Scoring PRotein INTeractions) (Li and Ilie. 2017), domain interaction, the semantic similarity of Gene Ontology (GO) terms (Ashburner et al., 2000), and co-participation in metabolic pathways.

We modified the original stacked generalized scheme (Džeroski and Ženko, 2004) using the predictions derived from the aforementioned detection methods to build the numerical features matrix (rows are the PPIs and columns are the predictions) used as input by the meta-classifier. Thus, we can combine multiple pieces of evidence on the interaction probabilities according to each detection method’s perspective.

The detection method based on semantic similarity is grounded on the assumption that proteins sharing functional annotations have a high chance of interacting (Jain and Bader, 2010). Hence, we performed a comparative analysis among five semantic similarity metrics (see Supplementary Material S1). Thus, we implemented the Pekar metric (Pekar and Staab, 2002) in the workflow to calculate three numerical features from the cellular component, biological process, and molecular function branches.

In addition to using functional annotation features, we also used protein structural information based on the possible interactions between conserved regions of their structure, such as domains, using data from the 3DID (three-dimensional interacting domains) database (Mosca et al., 2013). Let *D* = {*d*_1_, *d*_2_, …, *d*_*n*_} represent the list of all possible domains that can be annotated for proteins, *LDC* = {(*d*_1_, *d*_3_), (*d*_2_, *d*_3_), …, (*d*_*n*−1_, *d*_*n*_)} define a list of all known pairs of this annotation in 3DID and *LDP* = {(*d*_2_, *d*_4_), (*d*_1_, *d*_2_), …, (*d*_*n*−1_, *d*_*n*_)} represent the list of all pairwise combinations of the domains associated with proteins belonging to PPIs under evaluation. Then, the domain score is calculated using Eq. 1, defining a Jaccard index representing the domain pairs derived from the combination of the known interactions list of protein domains.

$$score_{Domain}=\frac{\left|LDC\;\cap \;LDP\right|}{\left|LDC\;\cup \;LDP\right|}$$ (1)

Finally, we further developed a method that considers the functional relationship between a pair of enzymes in the context of metabolic pathways. For this method, a list of all enzymes and their respective pathways in which they participate is retrieved from the KEGG (Kanehisa et al., 2016) database. Supposing a PPI between proteins A and B, let *V*_*A*_ = {*V*_1_, *V*_2_, …, *V*_*m*_} represent a list of all metabolic pathways associated with protein A and *V*_*B*_ = {*V*_1_, *V*_2_, …, *V*_*m*_} is a list of pathways related to protein B. This method’s pathway score is calculated following Eq. 2, which is a Jaccard index representing the fraction of pathways shared and participated in by the proteins in the PPI.

$$score_{Pathway}=\frac{\left|V_{A}\;\cap \;V_{B}\right|}{\left|V_{A}\;\cup \;V_{B}\right|}$$ (2)

#### 2.1.3 Classification and Analysis

The final PredPrIn step executes a combined analysis of all numerical features or evidence calculated using the detection methods. A variance unit then normalizes the feature matrix (Noda, 2008) and uses it to input the meta-level classifier Adaboost (Schapire, 2013) algorithm that implements the boosting technique. This step also applies the 10-fold method of cross-validation for model selection. As a result, this step returns the positively predicted PPIs (in test mode) or the trained model (in training mode) with a report containing the main performance evaluation metrics (Hossin and Sulaiman, 2015), such as the accuracy, precision, recall f1-measure, confusion matrix, and AUC-ROC plots.

### 2.2 PPIVPro–Validation Process of Predicted PPIs

The PPIVPro has two filtering modules (Supplementary Figure S3) to evaluate newly predicted PPIs, namely, 1) cellular co-localization filtering and 2) PPI extraction from scientific publications.

#### 2.2.1 Cellular Co-localization Filtering

We constructed a database of association rules (Hipp et al., 2000) using the co-occurrence of cellular components in the known validated interactions from the HINT database (Das and Yu, 2012). Then, we applied the Apriori algorithm (Hipp et al., 2000) according to the cellular component annotations iteratively assigned to HINT proteins to generate the association rules using an evaluation function as a stop criterion of the process. This function evaluates whether a subset of main cellular components is included among the rules. After this iterative process, the rules database contains cell location sets that presented high co-occurrence frequency and correspond to a double-column file (antecedent and consequent).

Considering a given PPI, the filtering module analyzes whether the two proteins’ cellular components are found in the antecedent and consequent columns in the same rules. Then, this proteins pair is returned as positive by this module.

#### 2.2.2 PPI Extraction From Scientific Publications

This PPIPubMiner module uses HGNC symbols (Povey et al., 2001) associated with the PPIs’ protein identifiers under evaluation as bait to filter the most relevant articles indexed in the PubMed^4^ and PubMed Central^5^ databases. The content of these papers is further retrieved using the NCBI API^6^ and stored in a knowledge base of processed xml files.

A cleaning step handles these files to remove the markup language tags, such as sections unrelated to the essential text in the article body paragraphs. This step returns the processed text of the sentences found in the abstract and main body of the papers.

Among all existing natural language processing (Manning et al., 2014) techniques to handle and prepare textual data, PPIPubMiner executes the extraction of sentences and tokens, word normalization to lower case, stemming, removal of stop words, and prioritization of verbs and nouns using part-of-speech tagging. These steps optimize the text mining and filtering of those sentences that have an interaction context.

We developed a context filtering dictionary to exclude terms (or sets of terms) that appear in the same sentence of proteins but are related to other regulatory events not directly associated with PPIs.

Another step of this module further checks the existence of protein entities^7^ in the filtered sentences. We also developed an entity recognizer to obtain evidence of experimental validation methods, such as entities found in the molecular interaction ontology.^8^

If the target proteins are found with verbs and nouns indicating an interaction context (for instance, signaling and binding), the final step generates a rule-based report for each protein pair. This report includes the sentences, the interacting words found, the proteins, and the experimental methods.

### 2.3 Datasets for Performance Assessment

We prepared six balanced datasets (described in Supplementary Table S8) to test the efficiency of PredPrIn parallel execution and compare its performance against related works. Each dataset contains 200 thousand PPIs, with 50% positive and 50% being negative. The positive protein pairs of validated group datasets were formed by interactions from DIP^9^ (2,469 PPIs), HPRD^10^ (12,094 PPIs) and Biogrid^11^ (85,437 PPIs) databases. The other group interactions was extracted from STRING^12^ database. We considered variations in the range of confidence scores in the STRING group to achieve protein pairs with a diversity of functional annotations and diminish the prediction evaluation bias. These first six datasets were named as the low score version^13^, since their negative pairs were retrieved from STRING using score less than 400. We also prepared a duplicate dataset, named as the random pairs version^14^, with the same positive pairs as the six aforementioned datasets. Still, all the negative pairs were randomly chosen among the available protein identifiers on the Uniprot database. The only restriction applied to these negative pairs was their absence in the known positive set.

These twelve datasets were used to test scalability and efficiency. The performance of trained models derived from these datasets was also evaluated on disease-state PPI prediction with a curated lung cancer PPI network^15^ (Li et al., 2017).

Only validated group datasets (low score version) were used for new PPIs prediction. In contrast, the STRING group datasets (low score version) were used to evaluate whether a model from inferred PPIs can predict PPIs from other related databases such as FunCoup (Persson et al., 2021), HumanNet (Hwang et al., 2019) and genemania (Franz et al., 2018). To assess the hypothesis mentioned above, we extracted and compiled^16^ all the genemania datasets of Physical interactions (202 datasets), all the HumanNet PPI datasets and the Funcoup PPIs with a confidence score above 0.900.

### 2.4 Datasets and PPI Prediction Methods Used for Performance Comparison

The performance comparison with other PPI prediction tools followed the same strategy used by the authors of Metago (Chen et al., 2019), where they compiled the reported scores of the prediction methods (PPI-MetaGo (Chen et al., 2019), PRED_PPI (Guo et al., 2010), TRI_tool (Perovic et al., 2017), hierarchical vector space model (HVSM) (Zhang et al., 2018), go2ppi (Maetschke et al., 2012), GIS-MaxEnt (Armean et al., 2018) and DeepSequencePPI (Gonzalez-Lopez et al., 2018), and executed PPI-Metago prediction experiments using the same datasets^17^ reported by these tools.

The species used in our analysis (Supplementary Table S10) were *Saccharomyces cerevisiae* (datasets SC1, SC2, SC4, SC5, and SC6), *Homo sapiens* (datasets HS1, HS3, HS4, and HS5), *Escherichia coli* (datasets EC1 and EC2), *Drosophila melanogaster* (datasets DM1 and DM2), *Caenorhabditis elegans* (dataset CE), *Schizosaccharomyces pombe* (SP), *Arabidopsis thaliana* (dataset AT) and *Mus musculus* (dataset MM).

### 2.5 Dataset for Predicting New Candidate PPIs

We designed a dataset with new candidate PPIs to test the models trained with PredPrIn for new PPIs discovery. We retrieved data from the Network of Cancer Genes (NCG) database (Repana et al., 2019), which contains two lists corresponding to known and candidate cancer genes. Hence, the PPI dataset was developed by collecting the proteins associated with these genes and applying a pairwise combination of the proteins in both lists. We generated approximately 800 thousand PPIs^18^, and we separated them into four datasets to be processed in parallel in PredPrIn. We obtained the processed annotations and the corresponding matrix of numerical features, which contained *M* lines related to the PPIs and *N* columns of calculated features provided by the detection methods.

## 3 Results and Discussion

### 3.1 PredPrIn Prediction Evaluation

#### 3.1.1 Assessment of Prediction Efficiency and Scalability

We compared the PredPrIn efficiency with the DPPI method (Hashemifar et al., 2018) using the same PPI dataset of the primary evaluation containing 50,000 protein pairs. We took the PredPrIn running times with and without using the Knowledge base (kb) to perform this comparison (Supplementary Table S9). The experiment was performed in a computer with 16 GB of RAM memory, 1 TB of hard disk, using Ubuntu 16.04 as operating system. The time corresponding to DPPI only considers the prediction and does not involve data preprocessing, which is the most time-consuming step for DPPI, especially in protein profile generation. For the same number of protein pairs, using the KB information, we improved the running time to less than 5 hours relative to DPPI, demonstrating the importance of the KB for experiment acceleration. Furthermore, almost 95% of the decreased time affected the preprocessing step, which was expected since the knowledge base mainly affects this step.

Regarding scalability, we performed the main experiment to evaluate the PredPrIn predictions and the workflow architecture for parallel execution of the six datasets of each version at a time (described in 2.3). We also indicate that the knowledge base for this experiment contributed to decreasing the running time from 55 to 47 h. Despite the considerable increase in the dataset size (50,000 to 1,200,000 PPIs), the additional execution time was not proportional, which means that the parallelism provided by the workflow architecture allowed our approach to be scalable. We estimated that the individual parallel processes assigned to each dataset had a maximum usage of RAM memory up to 2GB, which happens only in the SPRINT execution to obtain the sequence feature scores, then the usage decrease to 800 MB. Previous works related to the large-scale prediction of PPIs have not considered the strategy of distributing dataset load in a workflow architecture, and optimizing features acquisition and extraction by reusing prior computed information (Chen et al., 2008; Pan et al., 2010; You et al., 2014; Zhang et al., 2014). Some do not consider the preprocessing step in the running time evaluation (You et al., 2014; Hashemifar et al., 2018) while PredPrIn distributes the dataset load since this first step.

We enhanced the first PredPrIn step, including triggers (controller and reuse of the KB) to increase the speed in future prediction experiments. Hence, we improved the user experience by providing automatic feature extraction like previous works (Gonzalez-Lopez et al., 2018; Chen et al., 2019; Yang et al., 2020), and added more refinements to the preprocessing step. Furthermore, by using RDF (Cyganiak et al., 2014) data, we ensure that the preprocessing is flexible to the inputs required by other detection methods added to the numerical feature generation step in the future.

The PredPrIn prediction performance on predicting disease-state PPIs (Supplementary Material S2) was also assessed with the OncoPPI dataset (Li et al., 2017) containing 347 interactions. PredPrIn was used in the test mode with these interactions with each of the twelve models, the overall execution time took 8 min. According to the recall results, our tool reaches up to 87% of recall even without using specific information about the disease-state context.

#### 3.1.2 Comparison of PredPrIn Against Individual Detection Methods

We compared the prediction performance of the individual methods (Section 2.1.2) with PredPrIn using accuracy and F1-score comparison plots, one for each dataset. The models in each version (low score and random pairs) presented similar performance, so we chose only the plot for dataset six to demonstrate the results (Supplementary Figure S4) for each dataset version. PredPrIn obtained the highest accuracy and F1-score values in all datasets, ranging from 0.95 to 0.97 in low score version, and from 0.979 to 0.996 in random pairs version. The results show a better performance of individual methods in the random pairs version of the datasets, which also contributed to increase the scores for PredprIn. Hence, this result reinforces the statement that the combination of multiple pieces of evidence provided by PredPrIn yields better predictions than individual detection methods (Chang et al., 2016).

Interestingly, the detection methods based on semantic similarity (GO-CC, GO-MF, GO-BP) also have high accuracy values. However, the values are not larger than 0.80. This finding implies that our comparative analysis (Supplementary Material S1) of semantic similarity metrics to select the best metric was reflected in the excellent performance of this detection method. Moreover, the behavior observed in the accuracy and F1 score plots of the detection methods shows the importance of using multiple evidence for prediction. This trend was also demonstrated in previous related work (Kotlyar et al., 2015).

We also carried out several biological analyses (Supplementary Material S3) and confirmed the hypothesis related to Gene Ontology functional enrichment when comparing positively and negatively predicted PPIs. These biological analyses also demonstrated that the predicted PPIs conserve biological properties according to known parameters explored in the literature, such as the roles of hub proteins as proteins with high betweenness centrality.

#### 3.1.3 Comparison of PredPrIn With Known PPI Prediction Methods

We compared the PPI predictions with seven recent tools, and was evaluated in different datasets of several species (as described in Section 2.4). We executed PredPrIn in training mode to obtain the classification metrics report and we selected accuracy and precision metrics to evaluate PredPrIn against these tools. Besides PredPrIn was designed to perform large-scale predictions in parallel, these datasets are significantly smaller than those we built for the scalability and efficiency assessment described in Sections 3.1.1, 3.1.2. The more extensive dataset (SC6) used in the present section is unbalanced (just as SC4 and HS5), and it has 17,257 positive and 48,594 negative protein interactions.

**FIGURE 2:**
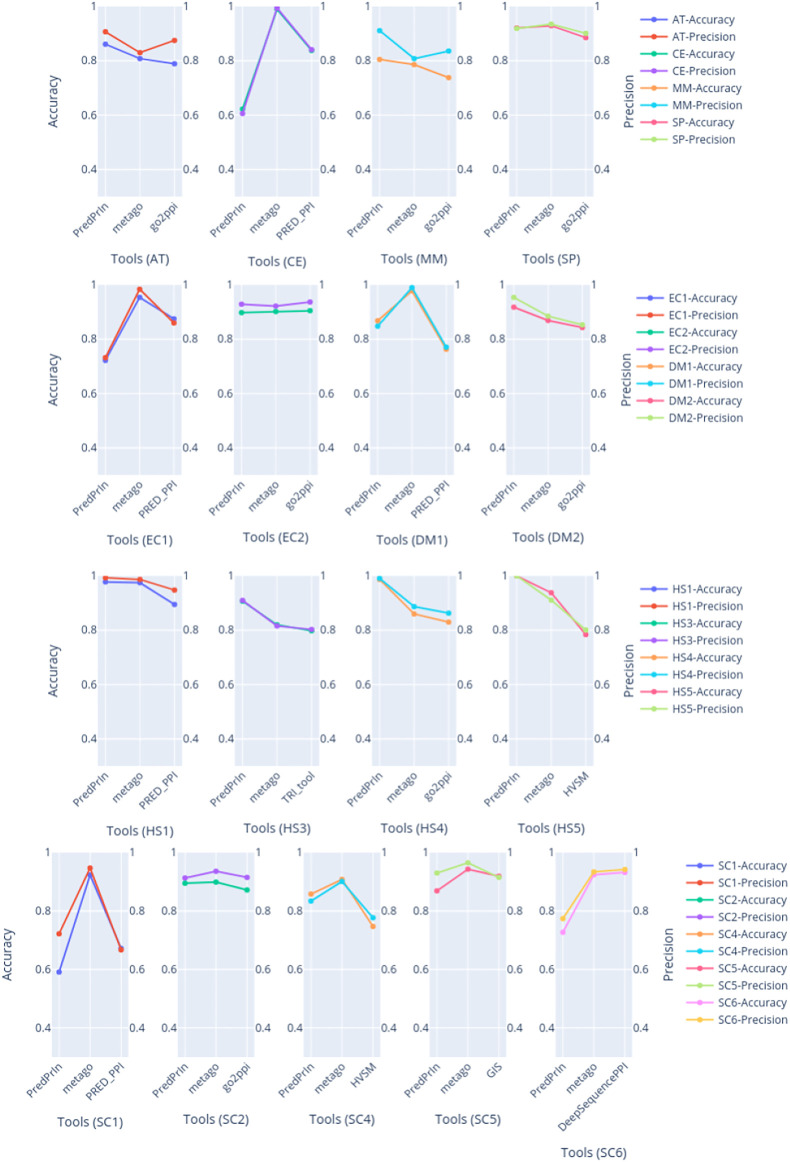
The plots are grouped by dataset, compared with accuracy and precision metrics between PredPrIn and the other tools according to each species datasets. The left y axis represents the accuracy values, and the right y axis refers to the precision ones. The colors are repeated according to each line of plots. The first line of plots are the isolated datasets for AT, CE, MM and SP species, the second line are the plots for the pairs of datasets belonging to DM and EC, the third line contains the plots for four datasets belonging to HS species. Finally, the last line represents the plots of the five datasets of SC species.

PredPrIn had the highest values of accuracy and precision for all human datasets (Figure 2), the detailed values of these metrics are described in Supplementary Table S11. This fact happened because we mainly designed PredPrIn to predict PPIs for the human organism. The core of the PredPrIn’s SPRINT predictor component was kept with the trained model for human proteins. A second factor to be considered is that the comparative analysis to select the most efficient semantic similarity metric was also performed for human PPIs. Besides PredPrIn being designed for humans, only two datasets (EC1 and CE) PredPrIn had accuracy under 72%. This means that the other new predictors added in the second step of PredPrIn leveraged and helped the PPI prediction of non-human organisms. PredPrIn metrics values are closer to the other tools like PPI-MetaGo with a difference under 0.180 between accuracy and precision in most datasets. We also surpassed the other tools in the datasets AT, DM2 and MM for both metrics.

PredPrIn and MetaGo use a stacked generalization architecture, and our results showed that this technique has great potential for PPI prediction. We developed PredPrIn to achieve compelling predictions without requiring a specific computational architecture, such as a graphic processing unit (GPU). PredPrIn combines automatic feature extraction and acquisition, reusing this information to optimize further experiments involving proteins already analyzed. DeepSequencePPI is the only tool used in this section analysis that offers automatic feature extraction from the raw sequence. Still, it does not reuse prior computed information when generating features for future experiments. PPI-MetaGo also computes the features but it requires hand-crafted protein annotations and sequences from the user in each experiment. PredPrIn added three new predictors to the base level of the stacking ensemble technique. At the same time, PPI-MetaGo uses sequence, go annotations and network-based features, using them in four classic classifiers of the base level. The total computation of the final score in its strategy increases according to the number of PPIs received as input.

#### 3.1.4 Prediction Analysis of Models Derived From Computationally Inferred PPIs

The traditional training models are computed from validated or literature-curated PPIs. However, the STRING database concentrates on submissions of predicted PPIs from several sources and prediction methods. This database has more than two trillion PPIs from 5090 organisms (Szklarczyk et al., 2018). Thus, the number of validated PPIs is significantly smaller than the predicted ones. The HINT database (Das and Yu, 2012) aggregates validated PPIs from eight curated databases. The total number of binary interactions is 164448 considering the 12 organisms in this database. Thus, this section’s analysis aims to evaluate the prediction performance using trained models only from the STRING datasets (described in Section 2.3). We also evaluated the impact of different ranges of confidence scores in the prediction to determine whether they reflect in the prediction performance.

**FIGURE 3:**
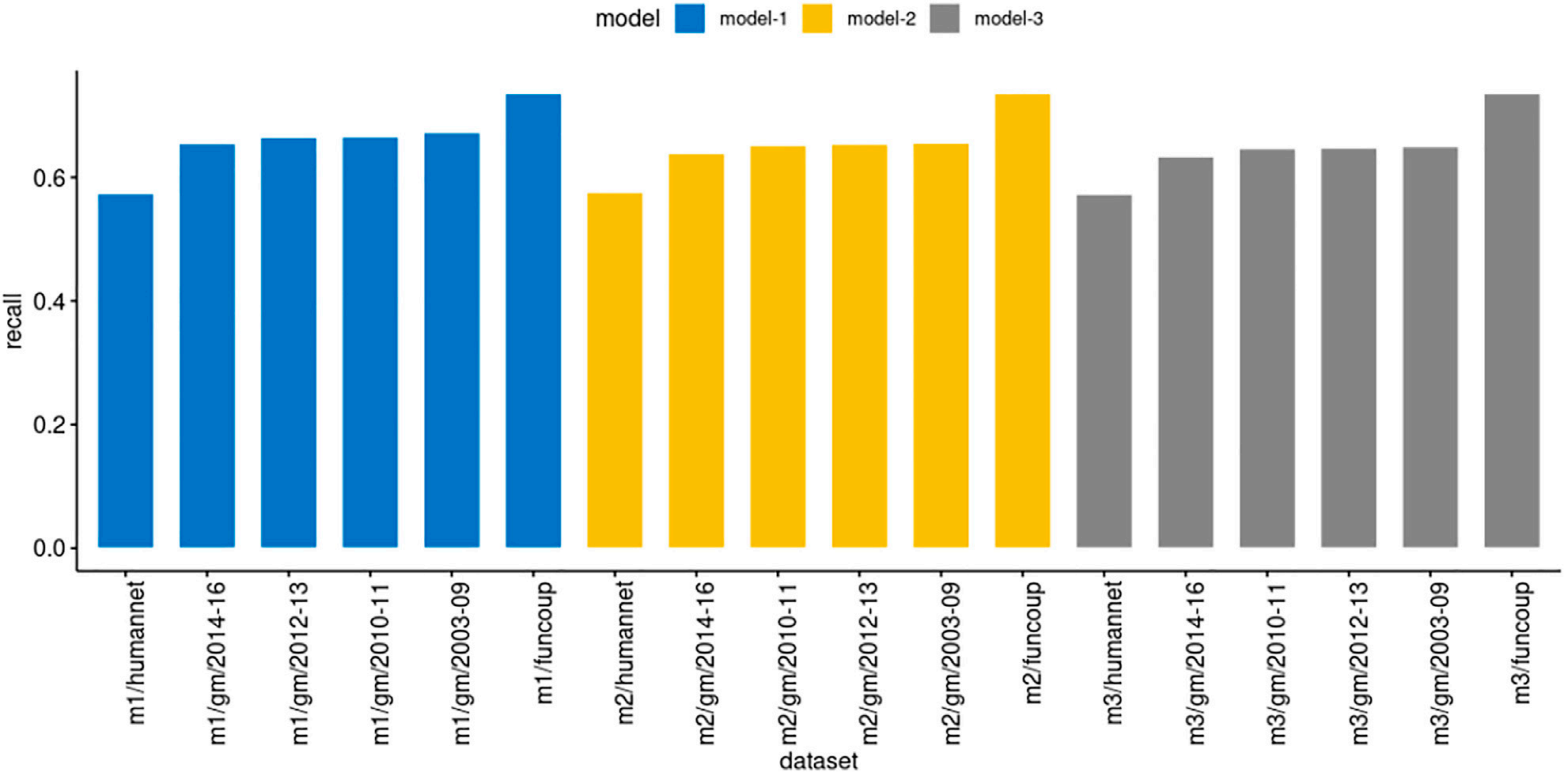
Comparison of recall in the models derived from DS4 (model3/m3), DS5 (model2/m2) and DS6 (model1/m1) trained by PredPrIn. The genemania datasets are organized in the following year intervals: 2003–2009 (51 datasets), 2010–2011 (46 datasets), 2012–2013 (44 datasets) and 2014–2016 (53 datasets). The standard deviations for the same intervals, respectively, for model 1 were 0.106, 0.145, 0.116, and 0.125, for model two were 0.107, 0.148, 0.108, and 0.121, finally, for model 3 were 0.109, 0.151, 0.104, and 0.122.

We executed PredPrIn in a test mode with the trained DS4, DS5, and DS6 against the datasets FunCoup, HumanNet and genemania. We evaluated using the recall metric to measure the ratio between positively predicted PPIs from PredPrIn and all possible true interactions from each database. Since all the physical interactions from genemania return 204 datasets, we organized the recall results for the three models grouping these datasets in year intervals (Figure 3). Due to the large-scale processing capability of PredPrIn, we processed all these datasets setting ten independent processes (10 datasets in parallel), the total execution time was 2 days and 17 h. All the models produced recall values varying from 57.3 to 73.6%. The lowest values occurred in the HumanNet and the highest ones in the FunCoup database. There were minor differences according to the confidence score of the PPIs in the models. Model 1 was trained with PPIs with the highest confidence scores and for all datasets produced the best recall values (67.3% for genemania sets, 73.6% for FunCoup and 57.4% for HumanNet), the difference between model 1 and two was higher (0.014, approximately) than the difference between model two and 3 (0.006).

**FIGURE 4:**
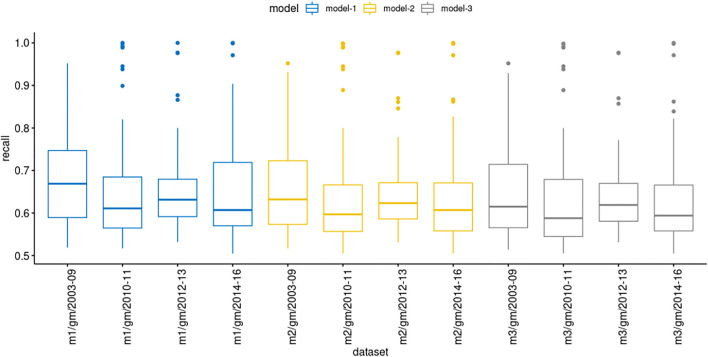
Comparison of recall values showing the mean location and distribution of recall values in each group of genemania datasets.

We evaluated the model prediction in the genemania datasets with more details to explore their recall values (Figure 4). Interestingly, in some datasets, highlighted as the outliers dots, the recall values were above 80%, besides the mean remained between 60 and 70%. Although our results showed that models derived from STRING (computationally inferred PPIs) showed a high recall performance in selected datasets, most of them, the prediction was unsatisfactory considering a cutoff value of 75%. We also observed that the confidence score of the PPIs in models produced low improvement on forecast.

PredPrIn enabled a screening analysis of models derived from inferred PPIs in more than 200 human datasets belonging to other functional databases. We evaluated STRING using only computationally inferred PPIs. In contrast, some works use in their methods only interactions with a high level in the experimental and literature-curated scores that forms the total combined score (Das and Chakrabarti, 2021; Ding and Kihara, 2019), limiting the power of the STRING database. Our results showed that STRING could find protein associations, and the models can be improved using this database in combination with another source of features. This strategy was used in the SDN2GO method (Cai et al., 2020) to predict protein function, in which they used primary sequence, annotations, network data (STRING) and domain information.

### 3.2 Evaluation of the Prediction of New PPIs

We experimented PredPrIn testing the models trained with PredPrIn (Section 3.1.2) for the validated group datasets against new candidate protein pairs (described in Section 2.5).

Using the models trained by PredPrIn in the numerical features of the new dataset, the predictor returned 5150 positive PPIs^19^. These interactions were submitted to the validation process proposed in Section 2.2. We developed this validation method to help curating PPIs, since most experimental validations are limited by the availability and cost of antibodies to recognize the proteins of interest just as the requirement of tagging novel proteins (Miteva et al., 2013). Regarding the use of *in-silico* methods to validate PPIs, some methods (Tan et al., 2004; Antony et al., 2008) proposed an approach to validate interactions based on the principle of coevolution. Specifically, one study (Antony et al., 2008) tested their approach on PPIs retrieved from text mining in a specific context for articles, including those belonging to “multiple sclerosis” terms. Compared with the cited study, the first filtering step of our validation process attempts to exclude PPIs in locations that invalidate the interaction more quickly than running sequence aligners such as blast (the most used tool in evolutionary methods). Furthermore, our approach searches the most relevant articles in any context that contain a relation between the target PPIs. We also attempt to recover the mentioned experimental assays in these articles to enrich the confidence of the exported report.

Concerning the results of our validation process, the first step (Section 2.2.1) filtered PPIs located in cell sites which has no channel to enable physical interaction. This step returned 4820 PPIs^20^ that were further evaluated by the second filtering step with the PPIPubMiner module (described in Section 2.2.2). Among the 330 removed protein interactions, most of these pairs were composed by one protein in the nucleus and the other in the extracellular matrix. In other cases of removed protein pairs, one or both proteins had zero or few general annotations about their location.

From the remaining 4820 pairs, we have found that 20 predicted interactions was already published in validated PPI databases with overlapping: DIP (2), HPRD (2), HINT (7), Biogrid (13). In STRING, we have found 30 interactions with score above 900, 2 with score between 800 and 900, and 14 with score ranging from 700 to 800.

As a result of the PPIPubMiner module, we found 3729 PPIs^21^ (out of 4820 remaining of the previous step) in published scientific papers that mention proteins and 50 protein pairs^22^ in an interaction context in one or more sentences in different articles. These final protein interactions provided by PPIPubMiner passed through manual curation to study the properties of these indicated interactions. The goal of this manual validation was analyzing the sentences retrieved for the 50 pairs at the end of the validation process. We evaluated each paper to check whether the interactions were really confirmed.

Regarding the use of text mining for PPI identification, most methods (Papanikolaou et al., 2015) start extracting from an article any protein pair in the PPI context instead of prioritizing the validation of specific protein interactions. Our approach is designed for more focused research, by selecting evidence in the most relevant papers indexed in Pubmed to validate and confirm the relationship of the predicted PPIs. We also build a knowledge base to optimize the extraction of information, by saving preprocessed sentences indexed by their origin. Also, context filtering reduces the number of false-positive sentences. The manual curation showed that this context-based filtering excluded protein pairs related to regulation events (mainly in exome and gene profiling studies).

According to the manual validation results, among the 50 pairs with sentences, two were already confirmed in Biogrid (LRIG3-GAL3ST1 and SMAD2-ZEB2 (also in HINT)). We also computed the occurrence of three cases: finding sentences with any interaction context, finding evidence of physical PPIs in the sentence and finding sentences out of interaction context (the main error).

There were only nine occurrences of unique sentences in which there was no interaction context, and the tool wrongly classified the sentences based on a gene expression and regulation context besides finding key interacting verbs like recruit and bind. These errors happened for the pairs APC-TCF7, HOXA13-HOXA10, JUN-ESRRG, JUN-GNA12, JUN-NR3C2, JUN-TLR7, JUN-TLR9, LMO2-SCAF4, ROBO2-CD200, and ROBO2-DLL1. Besides there was no evidence for APC-TCF7, this PPI was also predicted in STRING with a score above 900, which is a false positive.

For almost all the 50 pairs, PPIPubMiner identified interaction context (41). Among these 41 cases, 12 were confirmed as evidence^23^ of physical interaction of protein pairs predicted by PredPrIn, which are AR-TACC1, CCR4-YTHDF2, IL2-IL1B, IL2-CCKBR, IL2-TLR4, HMGA1-AURKA, DDX3X-MAPKAPK5, IRIG3-GAL3ST1, SMAD2-ZEB2, MYC-CEP170, NUP98-DOT1L, and RAF1-LGALS1. We highlight that, except for IRIG3-GAL3ST1 and SMAD2-ZEB2, all these interactions are not described in any PPI curated databases.

The other 28 cases were related to transcriptional interactions between proteins and gene promoters or some types of RNAs. Besides these last cases were not our primary focus while developing PPIPubMiner, the sentences of some of them also brought evidence of other physical PPIs^24^ involving the proteins of interest (not directly between them). Due to these repeated events, we intend to improve PPIPubMiner to classify the types of interaction that are retrieved in the sentences.

**FIGURE 5:**
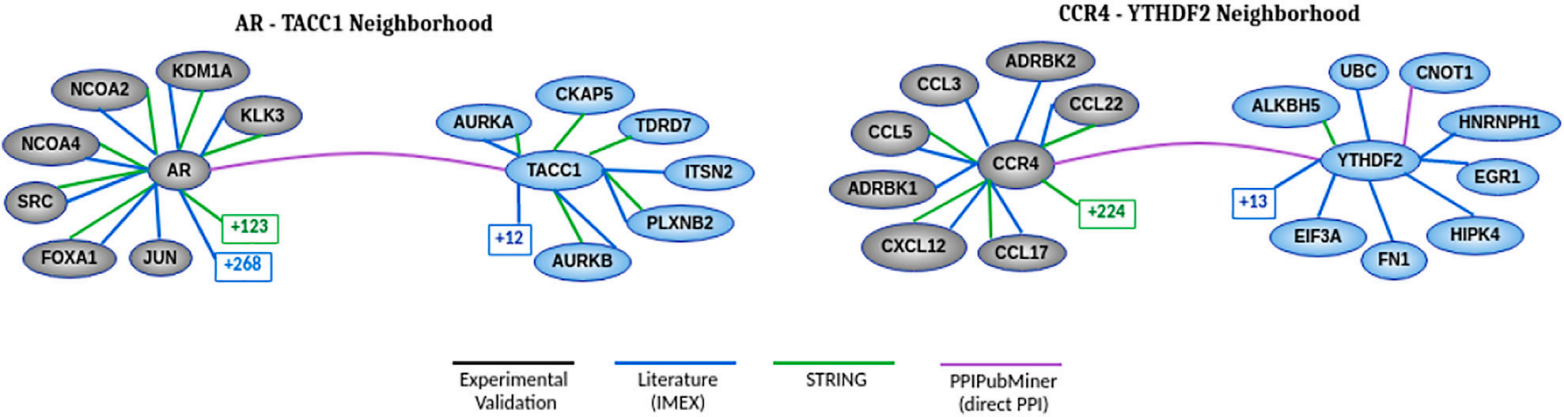
Neighborhood of each protein pair showing the relations between proteins according to each data source. The number found in squares represents the number of hidden nodes that we set apart to improve clarity. The gray vertices represent the proteins known to be associated with cancer. The blue vertices are candidate proteins for such an association. The white vertices indicate the proteins connecting the blue and gray vertices in more than one data source. The known interactions at the green edge were extracted from the STRING (Szklarczyk et al., 2018) database and we used a confidence score range above 900 to filter its interactions. The other known interactions were extracted from the experimentally validated (black edge) database (Rolland et al., 2014) and another one based on literature curation (Breuer et al., 2013) (IMEX - International Molecular Exchange Consortium, in blue edge). The PPIs of these two databases were retrieved using the *Network Analyst* tool (Zhou et al., 2019). The purple edge is based on our validation process.

At the end of this manual review, we selected two protein pairs to discuss in more detail. Figure 5 presents the known neighborhood of the two interactions using diverse PPI data sources. Our validation process expanded this known neighborhood by adding their relation according to the published articles returned as reports for each protein pair.

#### 3.2.1 Direct Interactions

The publication (Guyot et al., 2010) found by PPIPubminer confirmed that TACC1 has a role in controlling the transcription of nuclear hormone-receptors and nuclear locations. According to the results of experimental assays, TACC1 interacts physically with R*α*1, TR*α*2, and TR*β*1 in yeast and mammalian cells, and it also interacts with RXR*α*, RAR*α*, PPAR*γ*, ER*α*, GR, and AR. These last proteins are transcription factors belonging to two families of nuclear receptors.

We also found evidence (Du et al., 2016) supporting CCR4-YTHDF2. According to coimmunoprecipitation assays, these authors describe that YTHDF2 interacts with CAF1, CCR4A, and CNOT1 through the CCR4-NOT complex. This human complex consists of 9 subunits, including one structural subunit (CNOT1) and two catalytically active subunits (CAF1 and CCR4A). CAF1 presents a direct interaction with CNOT1, and CCR4A indirectly interacts with CNOT1 across CAF1. The interaction between YTHDF2 and CNOT1 is mediated by the SH (*Src homology*) domain. In addition to the interaction between CCR4 and YTHDF2, we also extracted the interaction between YTHDF2 and CNOT1, which was not included in HINT and Biogrid data sources.

## 4 Conclusion

PredPrIn provides a large-scale architecture to predict PPIs. We introduced new prediction methods based on domain and pathways. We also carried out a semantic similarity performance analysis to select the best semantic similarity metric. We also modified the stacking ensemble technique using the internal predictors as the base classifiers linking to a meta-level boosting classifier. This modification avoids the computation of the same features in many classic classifiers and decreases computing time. PredPrIn provides automatic feature extraction and reuses the processed annotations to accelerate the subsequent experiments. Many proteins are presented to PredPrIn less time it will take to execute the prediction. PredPrIn supports many datasets being processed at the same time. The user can define the available number of independent processes.

PredPrIn produced values of area under the curve above 90% for all six human datasets, and it also performed better than recent prediction tools in other human datasets. It was able to outperform in some non-human organisms. PredPrIn offers an infrastructure to perform large-scale and efficient predictions. The validation process can filter the new predicted interactions according to co-localization and text mining on biomedical literature, complementing the PredPrIn classification. We introduced a context filtering to avoid retrieving false positive sentences which is a significant problem in PPI literature extraction (Papanikolaou et al., 2015).

In summary, our workflow can efficiently and accurately predict binary protein-protein interactions and scale experiments with the flexibility to extensions in its features generation core. Furthermore, our validation process complements PredPrIn offering a way to execute post-processing with a focused search of possible interactions and evidence of them in relevant scientific publications.

## Data Availability

The datasets presented in this study can be found in online repositories. The names of the repository/repositories and accession number(s) can be found below: https://github.com/YasCoMa/predprin https://github.com/YasCoMa/ppi_validation_process.

## Footnotes

1
http://www.uniprot.org

2
http://pfam.xfam.org/

3
https://www.genome.jp/kegg/ko.html

4
http://pubmed.ncbi.nlm.nih.gov/

5
https://www.ncbi.nlm.nih.gov/pmc/

6
https://www.ncbi.nlm.nih.gov/books/NBK25498/

7
http://www.nactem.ac.uk/GENIA/tagger/

8
https://www.ebi.ac.uk/ols/ontologies/mi

9
https://dip.doe-mbi.ucla.edu/dip/Main.cgi

10
http://www.hprd.org/

11
https://thebiogrid.org/

12
https://string-db.org/

13
https://github.com/YasCoMa/predprin/blob/master/datasets_paper/version_low_score.zip

14
https://github.com/YasCoMa/predprin/blob/master/datasets_paper/version_random_pairs.zip

15
http://oncoppi.emory.edu/

16
https://github.com/YasCoMa/predprin/blob/master/datasets_paper/datasets_functional_prediction.zip

17
https://github.com/YasCoMa/predprin/blob/master/datasets_paper/datasets_multiTools_comparison.zip

18
https://github.com/YasCoMa/predprin/blob/master/datasets_paper/ncg_dataset.zip

19
https://github.com/YasCoMa/predprin/blob/master/results_new_pairs_prediction/supp_positive_pairs.tsv

20
https://github.com/YasCoMa/predprin/blob/master/results_new_pairs_prediction/filtered_pairs_by_assocrules.tsv

21
https://github.com/YasCoMa/predprin/blob/master/results_new_pairs_prediction/literature_evaluation_pairs.tsv

22
https://github.com/YasCoMa/predprin/blob/master/results_new_pairs_prediction/processed_sentences.zip

23
https://github.com/YasCoMa/predprin/blob/master/results_new_pairs_prediction/compiled-direct-interactions-predprin.tsv

24
https://github.com/YasCoMa/predprin/blob/master/results_new_pairs_prediction/compiled-direct-interactions-collateral.tsv
