# Supplementary material for: Large-Scale Protein Interactions Prediction by Multiple Evidence Analysis Associated With an In-Silico Curation Strategy
Source: Front Bioinform. 2021 Sep 6;1:731345. doi: 10.3389/fbinf.2021.731345 (PMC9581021; doi:10.3389/fbinf.2021.731345)
Supplement: Supplementary file 1 [file DataSheet1.PDF]

Supplementary Material

1 SUPPLEMENTARY MATERIAL 1 - COMPARATIVE ANALYSIS OF SEMANTIC SIMILARITY METRICS

The semantic similarity methods used in this study of comparative analysis were Resnik (Resnik (1999)), Lin (Lin et al. (1998)), Jiang (Jiang and Conrath (1997)), Pekar (Pekar and Staab (2002)) and Wang (Wang et al. (2007)). Semantic similarity is important for protein interactions prediction because similar objects tend to behave similarly and the graph structure of the Gene Ontology allows the comparison of GO terms from annotated gene products by semantic similarity (Pesquita (2017)).

The five chosen methods measure the semantic similarity between two GO terms, however, our interest is to calculate the overall similarity between proteins annotated with one or more GO terms. There are many methods (Yu et al. (2010)) to combine all the similarities generated between the pairwise combination of proteins GO annotations like the maximum, average, rcmax and bma. We chose bma to execute the combination because it is built with the best concepts of the other combination methods. BMA uses the Best-Match Average strategy and it calculates the average of all maximum similarities on each row and column (the combination of m terms of one protein in relation to the n terms of the other protein forms a $m \times n$ matrix).

The first phase of the study about these metrics was the development of six datasets, each one has 15 columns and 200,000 rows, that means the five methods calculated for the three GO branches (Biological Process (BP), Molecular Function (MF) and Cellular Component (CC)) for each candidate pair (200,000). In this dataset, 50% of these pairs were extracted from Biogrid[1] and HINT[2], while the other half from STRING[3] using confidence score ranging fro 100 to 200. The next phase was the values normalization for each column to the range of 0 to 1 and, then, calculate true positive rate and false positive rate for the ROC (Receiver Operating Characteristic) curve (Fan et al. (2006)), adding the area under curve (AUC) to use as final metric to judge which of the metrics was the best and stable for the GO branches in the six datasets.

The results are organized for each branch because they form three of the six evidence in PredPrIn, and we compared the performance to choose which metric have the high value of AUC for six datasets and branches. In the first dataset (results in table S1), for the GO biological process (BP) branch, the metric with best performance was Jiang followed by Pekar, which have AUC values 0.56 and 0.49, respectively. For the GO molecular function branch, the metric with best performance was Jiang followed by Pekar and

Table S1. Area under curve values for each metric in each branch for the first dataset

**Metrics**	GO BP	GO MF	GO CC
**Resnik**	0.48	0.47	0.49
**Lin**	0.48	0.51	0.47
**Jiang**	0.56	0.56	0.53
**Pekar**	0.49	0.52	0.49
**Wang**	0.48	0.52	0.49

[1] https://thebiogrid.org/
[2] http://hint.yulab.org/
[3] https://string-db.org/

Wang, which have AUC values 0.56, 0.52 and 0.52, respectively.
For the GO component cellular branch, the metric with best performance was Jiang and three other metrics (Resnik, Pekar and Wang) had the same AUC value which was 0.49. In this dataset Jiang and Pekar performed well for all the branches and are candidates to use inside for workflow.

In second dataset (results in table S2), for the GO biological process (BP) branch, the metrics with best performance were Resnik followed by Pekar, which have the same AUC value (0.60). For the GO molecular function branch, the metric with best performance was Wang followed by Lin, which have AUC values 0.66 and 0.65, respectively.

Table S2. Area under curve values for each metric in each branch for the second dataset

**Metrics**	GO BP	GO MF	GO CC
**Resnik**	0.60	0.59	0.63
**Lin**	0.59	0.65	0.61
**Jiang**	0.57	0.64	0.61
**Pekar**	0.60	0.64	0.63
**Wang**	0.59	0.66	0.63

For the GO component cellular branch, the metrics with best performance were Resnik, Pekar and Wang and they had the same AUC value which was 0.63. In this dataset Resnik and Pekar performed well for GO BP and GO CC but Pekar in GO MF was just 0.02 far from Wang while Resnik had a distance of 0.07, so Pekar was chosen as candidates to use inside for workflow.

In third dataset (results in table S3), for the GO biological process (BP) branch, the metrics with best performance were Resnik followed by Lin and Pekar, which have the same AUC value (0.60). For the GO molecular function branch, the metric with best performance was Wang followed by Lin and Pekar, which have AUC values 0.66, 0.65 and 0.65, respectively.

Table S3. Area under curve values for each metric in each branch for the third dataset

**Metrics**	GO BP	GO MF	GO CC
**Resnik**	0.60	0.60	0.63
**Lin**	0.60	0.65	0.61
**Jiang**	0.57	0.64	0.61
**Pekar**	0.60	0.65	0.63
**Wang**	0.59	0.66	0.63

For the GO component cellular branch, the metrics with best performance were Resnik, Pekar and Wang and they had the same AUC value which was 0.63. In this dataset Resnik and Pekar performed well for GO BP and GO CC but Pekar in GO MF was just 0.01 far from Wang while Resnik had a distance of 0.06 and Pekar was chosen as candidate to use inside for workflow.

In fourth dataset (results in table S4), for the GO biological process (BP) branch, the metrics with best performance were Resnik followed by Lin and Pekar, which have the same AUC value (0.67). For the GO molecular function branch, the metric with best performance was Wang followed by Pekar, which have same AUC value of 0.60.
For the GO component cellular branch, the metrics with best performance were Resnik, Pekar and Wang and they had AUC values of 0.65, 0.63 and 0.63, respectively. In this dataset, Resnik and Pekar also performed well for GO BP and GO CC and Pekar in GO MF had the same value as Wang (which was the

Table S4. Area under curve values for each metric in each branch for the fourth dataset

Metrics	GO BP	GO MF	GO CC
**Resnik**	0.67	0.59	0.65
**Lin**	0.67	0.59	0.61
**Jiang**	0.52	0.58	0.59
**Pekar**	0.67	0.60	0.62
**Wang**	0.66	0.60	0.62

best for this branch) while Resnik had a distance of 0.01 and Pekar was chosen as candidate to use inside for workflow.

In fifth dataset (results in table S5), for the GO biological process (BP) branch, the metrics with best performance were Jiang followed by Wang and Pekar, which have these AUC values: 0.71, 0.70 and 0.69. For the GO molecular function branch, the metric with best performance was Lin followed by Wang, which

Table S5. Area under curve values for each metric in each branch for the fifth dataset

Metrics	GO BP	GO MF	GO CC
**Resnik**	0.68	0.53	0.64
**Lin**	0.68	0.55	0.63
**Jiang**	0.71	0.54	0.63
**Pekar**	0.69	0.54	0.63
**Wang**	0.70	0.55	0.63

have same AUC value of 0.55.
For the GO component cellular branch, the metrics with best performance were Resnik with the AUC value of 0.64, while the others had a value of 0.63. In this dataset, each branch had different best metrics and neither of them was chosen as a candidate for this dataset.

In sixth dataset (results in table S6), for the GO biological process (BP) branch, the metrics with best performance were Jiang followed by Wang and Pekar, which have AUC value of 0.79, 0.79 and 0.78, respectively.

Table S6. Area under curve values for each metric in each branch for the sixth dataset

Metrics	GO BP	GO MF	GO CC
**Resnik**	0.77	0.63	0.72
**Lin**	0.77	0.65	0.68
**Jiang**	0.79	0.64	0.69
**Pekar**	0.78	0.65	0.70
**Wang**	0.79	0.66	0.70

For the GO molecular function branch, the metric with best performance was Wang followed by Lin and Pekar, which have AUC values of 066, 0.65 and 0.65, respectively.
For the GO component cellular branch, the metrics with best performance were Resnik, Pekar and Wang and they had AUC values of 0.72, 0.70 and 0.70, respectively. In this dataset, Wang and Pekar performed well in all three branches but Wang was the chosen candidate for this dataset.

Thus, analyzing the candidates assigned for each dataset, Wang and Pekar, the unique metric which maintained a stable performance behavior for all the datasets and GO branches was the Pekar. So it was picked to calculate GO BP, GO MF and GO CC evidences.

2 SUPPLEMENTARY MATERIAL 2 - ANALYSIS OF TRAINED MODELS IN LUNG CANCER PPI NETWORK

The trained models derived from the low score and random pairs version datasets were used to assess the PredPrIn performance on predicting validated disease-state PPIs associated to lung cancer disease. The proteins in a disease state may have mutations that changes their biding interface or causes biochemically dysfunctional allosteric changes (Gonzalez and Kann (2012)). These modifications may change their natural interactors and lead to the recruitment of others. The same way as in disease-state, the interactions in cell or tissue specific are better detected using the information of gene expression to quantify the availability of proteins in that context, and then filter the possible interactions (Yeger-Lotem and Sharan (2015); Bossi and Lehner (2009)). Besides our tool takes no gene expression data into account, we analyzed its performance to predict disease-state PPIs in a curated lung cancer protein interaction network (Li et al. (2017)). This network has 347 interactions and we computed the recall, which is the rate between the amount of PPIs that PredPrIn predicted as positive and these 347 true interactions. The results show that the models from random pairs version datasets had a better performance (with a recall difference of 0.2) than those from low score version datasets. Although PredPrIn does not take co-expression networks, conformational changes and specific sites data into account, our tool was able to reach up to 87% of recall, showing that even in specific conditions it is able to detect PPIs.

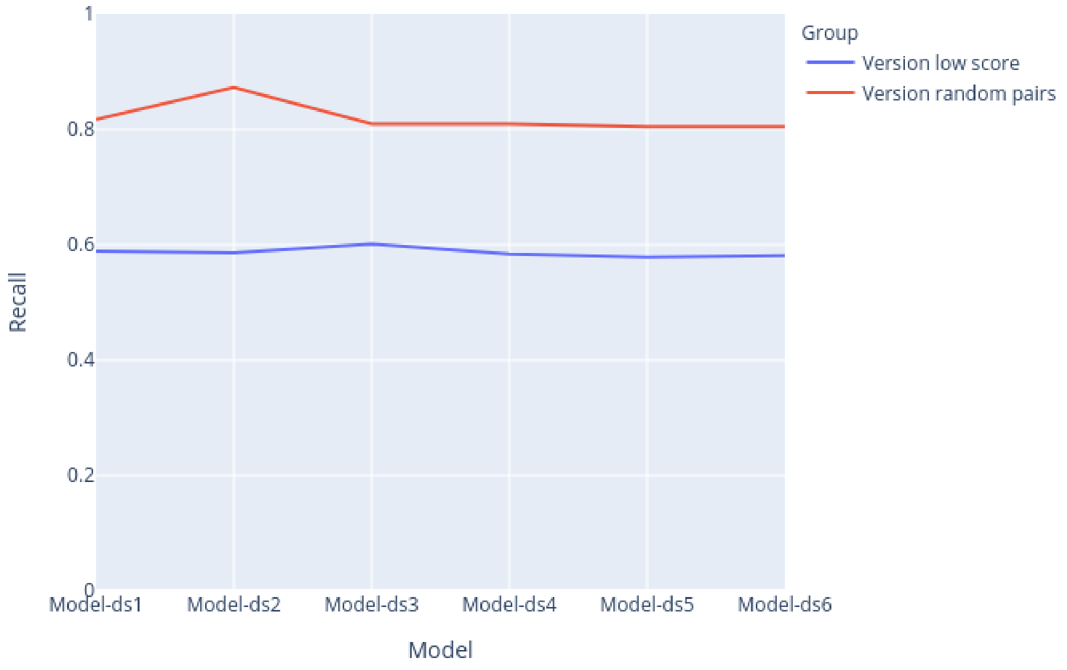

Figure S1. Performance of PredPrIn on predicting lung cancer PPIs using models derived low score and random pairs datasets.

3 SUPPLEMENTARY MATERIAL 3 - FUNCTIONAL ENRICHMENT AND TOPOLOGICAL EVALUATION OF PREDPRIN PREDICTED PPIS

3.1 Sharing of functional annotation terms

We evaluated the PPIs predicted by PredPrIn to verify whether they are consistent with the assumption that proteins are more likely to interact with others with similar functional annotations (Jain and Bader (2010)). For this analysis, we considered two cases for the functional annotations according to the Gene Ontology terms (Ashburner et al. (2000)). The first case corresponds to the sharing of biological process terms in contrast the second case concerns proteins sharing terms from three Gene Ontology branches at the same time (biological process, molecular function, and cellular component). We compared these cases in the PPIs predicted as positive or false for the six datasets in the experiment performed for computational evaluation (Section 3.1). Figure S2 shows the percentage of sharing in these two cases for the false or positively predicted pairs in each dataset.

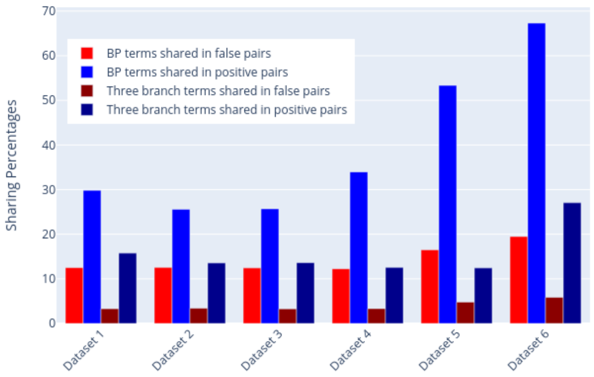

Figure S2. Percentages of GO biological process and the three branch terms shared in the pairs predicted as positive or negative in each dataset. The positively predicted pairs share more annotations in the two conditions than the false predicted pairs for all datasets, thereby reinforcing the assumption that proteins with similar functions have a high probability of interacting with each other.

The pairs predicted as positive shared more annotations on both cases than the pairs predicted as false for all six datasets, as expected according to the previously mentioned assumption. In the narrowest case, the positive pairs also shared more annotations than the false pairs. Since this part of the analysis involves a particular sharing operation, we expected that the percentage values would decrease. A previous work (Schoenrock et al. (2014)) performed a similar analysis evaluating the number of terms shared in the GO branches in known reported human PPIs and the ones predicted in their study. Our findings are consistent with their results for the same sharing cases, in which positively predicted PPIs have more similar annotations than random PPIs. This behavior was also previously observed in PPI networks of other species, such as yeast (Schwikowski et al. (2000); Von Mering et al. (2002)), in which interacting proteins were found to be more likely to share molecular functions and cellular components.

3.2 Analysis of the PPI network topology

The goal of this topological analysis is to compare the role of proteins with high values of centrality metrics, according to available information about PPI networks. In this experiment, the pairs predicted as positive were represented as a directed graph for each dataset and centrality metrics were used to rank the

proteins according to their degree and betweenness values (Koschützki and Schreiber (2008)). We further evaluated the biological processes of the top 10, 25, and 50 proteins in these ranked lists for these metrics. Table S7 summarizes the biological processes common among proteins in the three lists (top 10, 25, and 50) of each dataset.

Table S7. Most representative biological processes found in top 10, 25 and 50 nodes with highest degrees and betweenness centrality values.

**Dataset 1**							
**Biological Process**		**Top 10**		**Top 25**		**Top 50**	
		# proteins	P-value	# proteins	P-value	# proteins	P-value
**Hubs**	Regulation of nucleobase-containing compound metabolic process	8	4.00e-04	17	1.89e-06	30	1.63e-11
	Regulation of RNA metabolic process	8	3.86e-04	16	3.50e-06	29	1.93e-11
	Positive regulation of signal transduction	6	4.00e-04	11	7.42e-06	22	3.38e-12
**Best betweenness**	Regulation of cellular protein metabolic process	7	3.86e-04	14	4.27e-06	25	5.59e-11
**Dataset 2**							
**Biological Process**		**Top 10**		**Top 25**		**Top 50**	
		# proteins	P-value	# proteins	P-value	# proteins	P-value
**Hubs**	Regulation of nucleobase-containing compound metabolic process	9	5.13e-04	20	1.30e-07	39	1.63e-15
	Regulation of gene expression	9	7.01e-04	18	6.68e-06	38	5.91e-14
	Regulation of cellular biosynthetic process	8	1.89e-03	19	7.34e-07	38	1.97e-14
**Best betweenness**	Regulation of gene expression	10	3.49e-05	18	4.81e-06	36	1.23e-11
	Regulation of nucleobase-containing compound metabolic process	10	2.40e-05	19	5.92e-07	34	3.26e-11
	Regulation of cellular biosynthetic process	9	1.80e-04	18	2.38e-06	34	4.70e-11
**Dataset 3**							
**Biological Process**		**Top 10**		**Top 25**		**Top 50**	
		# proteins	P-value	# proteins	P-value	# proteins	P-value
**Hubs**	Regulation of nucleobase-containing compound metabolic process	9	7.70e-04	20	1.29e-07	39	9.98e-16
	Regulation of gene expression	9	7.70e-04	19	1.04e-06	38	4.34e-14
	Regulation of macromolecule biosynthetic process	8	1.77e-03	19	4.79e-07	37	1.61e-14
**Best betweenness**	Regulation of nucleobase-containing compound metabolic process	10	2.40e-05	19	2.72e-07	38	4.58e-15
	Regulation of gene expression	10	3.49e-05	20	1.61e-07	38	9.03e-14
	Regulation of cellular biosynthetic process	9	1.80e-04	18	2.13e-06	37	9.03e-14
**Dataset 4**							
**Biological Process**		**Top 10**		**Top 25**		**Top 50**	
		# proteins	P-value	# proteins	P-value	# proteins	P-value
**Hubs**	Positive regulation of phosphorylation	7	3.41e-06	14	1.07e-09	23	1.45e-14
	Positive regulation of phosphorus metabolic process	7	3.99e-06	14	1.07e-09	23	1.92e-14
	Negative regulation of protein metabolic process	8	1.04e-06	14	1.07e-09	22	1.72e-13
**Best betweenness**	Cellular response to organic substance	0	-	18	1.62e-09	34	8.44e-17
	Cellular response to chemical stimulus	0	-	20	2.35e-10	37	3.08e-17
	Regulation of protein metabolic process	0	-	18	2.07e-09	34	1.46e-16
**Dataset 5**							
**Biological Process**		**Top 10**		**Top 25**		**Top 50**	
		# proteins	P-value	# proteins	P-value	# proteins	P-value
**Hubs**	Membrane organization	7	4.62e-07	10	6.51e-06	13	2.60e-06
	Detection of stimulus	6	7.73e-06	8	6.53e-05	12	7.19e-06
	Sensory perception of taste	5	2.90e-08	6	1.79e-05	7	1.56e-07
**Best betweenness**	Response to stress	10	3.42e-06	21	8.73e-10	38	3.17e-15
	Cellular response to chemical stimulus	10	4.77e-07	22	3.02e-12	36	2.52e-16
	Response to endogenous stimulus	8	1.79e-06	17	1.99e-11	30	1.39e-18
**Dataset 6**							
**Biological Process**		**Top 10**		**Top 25**		**Top 50**	
		# proteins	P-value	# proteins	P-value	# proteins	P-value
**Hubs**	Viral process	8	1.10e-08	17	6.49e-16	33	1.64e-30
	Symbiont process	8	1.52e-08	17	1.88e-15	33	9.28e-30
	DNA metabolic process	9	9.53e-10	16	1.16e-13	27	8.22e-21
**Best betweenness**	Cellular response to stress	10	9.00e-09	23	2.49e-18	37	4.17e-24

Proteins with high degree centrality are usually known as hub proteins (Yu et al. (2007)). In this analysis, we considered these hubs with a global approach without differentiating them according to the types of hubs that may exist in biological networks (Agarwal et al. (2010); Wang and Jin (2017)).

The biological processes belonging to hub proteins are related to some regulatory pathways essential to the human organism. Some of these processes were involved in regulating metabolic processes, gene expression, cellular biosynthetic processes, and signal transduction. Some different processes found in datasets of the STRING group were phosphorylation, membrane organization, detection of stimulus, viral process, and symbiont process. As expected, the most frequent processes found for these hubs complied with essential functions for an organism (Yu et al. (2007)), which implies that the PPI network derived from positive predicted pairs by PredPrIn preserves the topological properties of known PPI networks. A previous work (Schoenrock et al. (2014)) analyzed their predicted PPIs using centrality metrics and found hub proteins performing the role of transcription and DNA-related regulation biological processes. They also reported signaling pathways in proteins with high betweenness values. We also found biological processes

for these proteins corresponding to signaling events of the cell response. Additionally, the aforementioned authors reported protein kinases acting as linkers (Gokhale and Khosla (2000)). In our analysis, these proteins were usually identified as hubs according to the phosphorylation biological processes (Ardito et al. (2017)). Another recent work (Buljan et al. (2020)) also showed that these proteins related to posttranslational modifications could act as hubs in PPI networks, thus supporting our results.

For dataset six, we also found that the viral and symbiont processes in proteins were detected as hubs for the three top lists. These processes are also highly enriched in virus-host protein interactions (Durmuş and Ülgen (2017)). The interactions predicted as positive in dataset six overlapped with protein pairs related to the virus-host interaction network.

The biological processes that pertain to proteins with high betweenness value are primarily associated with short paths between two proteins in different modules, thus representing chokepoints and helping to better establish communication in a biological network. Some of the processes were related to the regulation of metabolic processes, biosynthesis, and gene expression. Other different processes were associated with cellular responses to stress, organic substances, or endogenous and chemical stimuli. In summary, some processes are essential and others are associated with communication modules, which are compatible with the roles expected for proteins with high betweenness values (Gokhale and Khosla (2000)).

4 SUPPLEMENTARY TABLES AND FIGURES

4.1 Figures

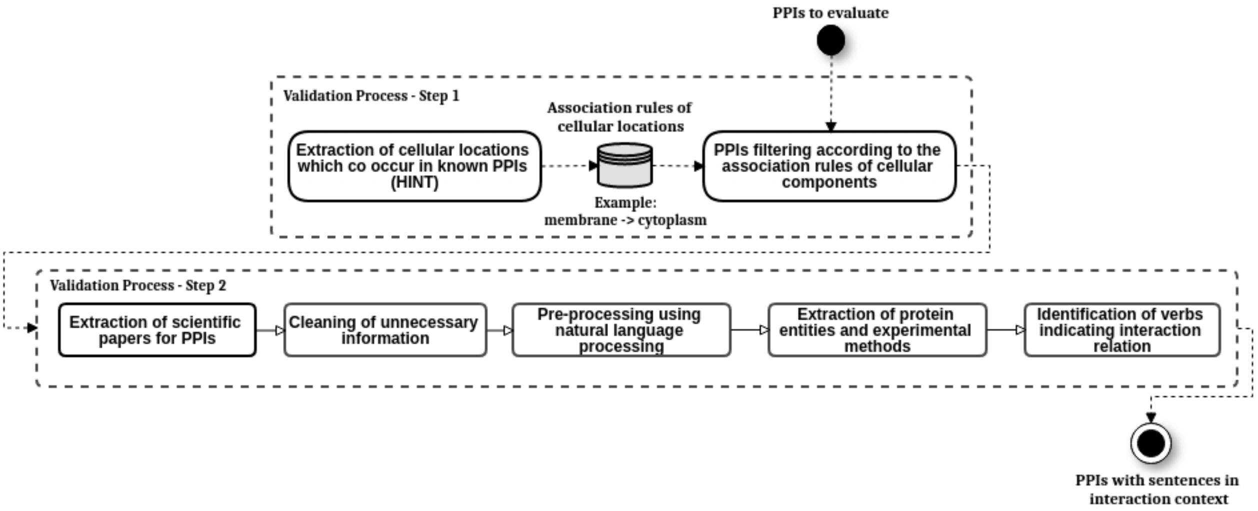

Figure S3. Diagram of validation process. Representation of the tasks composing the cellular co-localization filtering module (step 1) and the PPI extraction from scientific publications module (step 2).

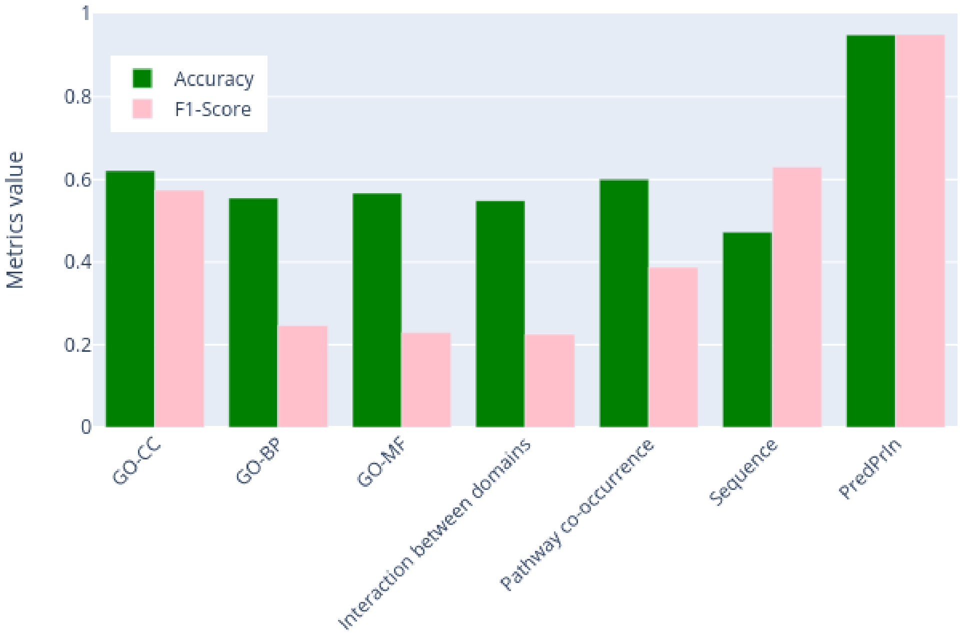

(a) Comparison for models from low score version datasets

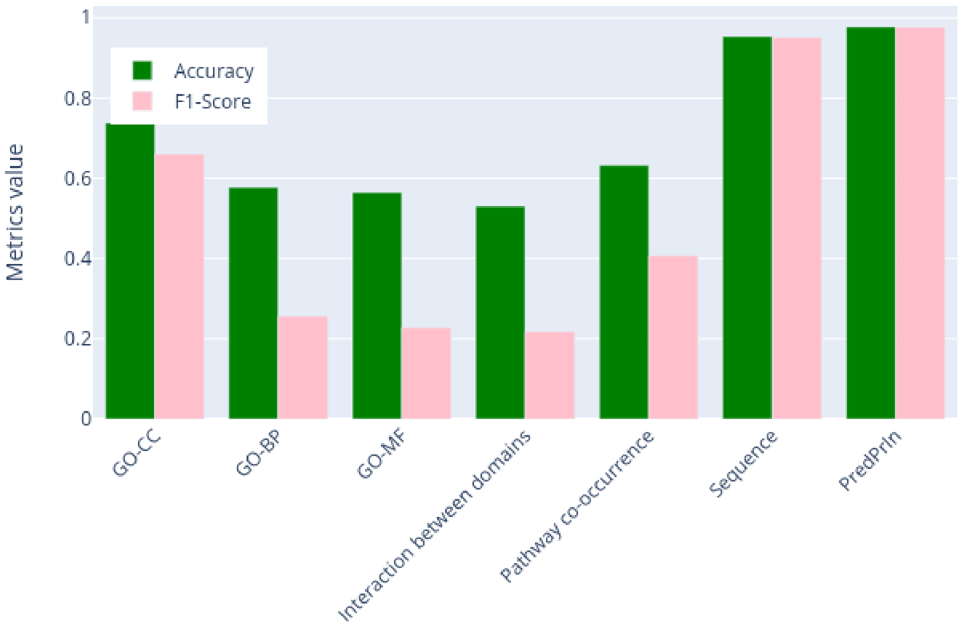

(b) Comparison for models from random pairs version datasets

Figure S4. Plots (a) and (b) show the accuracy and F1 scores for the individual methods and PredPrIn for the dataset six of the two datasets versions (low score and random pairs).

4.2 Tables

Table S8. Organization and composition of the datasets with negative pairs from STRING. Positive or negative pairs refer to those with high or low interaction probability, respectively. Three datasets (DS1, DS2, DS3) were organized in the validated cluster according to the positive pairs in the PPI reference databases (STRING, DIP, HPRD, and Biogrid). The other half of the datasets were clustered in the STRING group using different score ranges.

Group	Dataset	Sources		Score range	
		Negative	Positive	Negative	Positive
Validated	DS1	STRING	DIP, HPRD, BioGrid	100-200	-
Validated	DS2	STRING	DIP, HPRD, BioGrid	100-200	-
Validated	DS3	STRING	DIP, HPRD, BioGrid	100-200	-
STRING	DS4	STRING	STRING	100-200	700-800
STRING	DS5	STRING	STRING	200-300	800-900
STRING	DS6	STRING	STRING	300-400	900-1000

Table S9. Comparison of the execution time between the DPPI and PredPrIn methods with a dataset of 50,000 PPIs. In the PredPrIn case, we also show the time spent with a knowledge base (KB) and without it. The DPPI does not have this feature. The time is shown in hours.

Method	With KB	Without KB
PredPrIn	22	12
DPPI	-	17

Table S10. Datasets used for performance comparison with seven PPI prediction methods. The datasets were identified according to the species, and the prediction tools indicate that the specific dataset was firstly reported and used for evaluation by the respective prediction method. Most of these datasets are balanced except HS5, SC4 and SC6.

Identifier	Species	Proteins	Interactions (positive/negative)	Prediction Tool
EC1	Escherichia coli	1834	6954/6954	PRED_PPI
EC2	Escherichia coli	589	1167/1167	go2ppi-RF
DM1	Drosophila melanogaster	7059	21975/21975	PRED_PPI
DM2	Drosophila melanogaster	658	321/321	go2ppi-RF
HS1	Homo sapiens	9439	37027/37027	PRED_PPI
HS3	Homo sapiens	1515	12244/12244	TRI_tool
HS4	Homo sapiens	3296	3490/3490	go2ppi-RF
HS5	Homo sapiens	6037	1091/3427	HVSM
SC1	Saccharomyces cerevisiae	2245	3956/3956	PRED_PPI
SC2	Saccharomyces cerevisiae	3291	15238/15238	go2ppi-RF
SC4	Saccharomyces cerevisiae	5436	4529/10831	HVSM
SC5	Saccharomyces cerevisiae	454	500/500	GIS-MaxEnt
SC6	Saccharomyces cerevisiae	4424	17257/48594	DeepSequencePPI
CE	Caenorhabditis elegans	2640	4030/4030	PRED_PPI
SP	Schizosaccharomyces pombe	904	742/742	go2ppi-RF
AT	Arabidopsis thaliana	756	541/541	go2ppi-RF
MM	Mus musculus	1088	500/500	go2ppi-RF

REFERENCES

Agarwal, S., Deane, C. M., Porter, M. A., and Jones, N. S. (2010). Revisiting date and party hubs: novel approaches to role assignment in protein interaction networks. *PLoS Comput Biol* 6, e1000817

Ardito, F., Giuliani, M., Perrone, D., Troiano, G., and Lo Muzio, L. (2017). The crucial role of protein phosphorylation in cell signaling and its use as targeted therapy. *International journal of molecular medicine* 40, 271–280

Ashburner, M., Ball, C. A., Blake, J. A., Botstein, D., Butler, H., Cherry, J. M., et al. (2000). Gene ontology: tool for the unification of biology. *Nature genetics* 25, 25–29

Bossi, A. and Lehner, B. (2009). Tissue specificity and the human protein interaction network. *Molecular systems biology* 5, 260

Table S11. Accuracy and precision results of the PredPrIn performance compared with the other PPI prediction methods along the 17 datasets.

Tool	Dataset	Accuracy	Precision
PredPrIn	AT	0.861	0.907
PredPrIn	CE	0.622	0.606
PredPrIn	DM1	0.868	0.848
PredPrIn	DM2	0.918	0.954
PredPrIn	EC1	0.722	0.732
PredPrIn	EC2	0.898	0.929
PredPrIn	HS1	0.978	0.993
PredPrIn	HS3	0.907	0.910
PredPrIn	HS4	0.987	0.991
PredPrIn	HS5	1.000	1.000
PredPrIn	MM	0.805	0.911
PredPrIn	SC1	0.591	0.722
PredPrIn	SC2	0.895	0.913
PredPrIn	SC4	0.858	0.834
PredPrIn	SC5	0.869	0.930
PredPrIn	SC6	0.727	0.774
PredPrIn	SP	0.921	0.919
Metago	HS1	0.975	0.987
Metago	EC1	0.954	0.984
Metago	DM1	0.978	0.990
Metago	CE	0.990	0.995
Metago	SC1	0.923	0.947
Metago	HS2	0.910	0.834
Metago	HS3	0.820	0.816
Metago	SC2	0.899	0.936
Metago	HS4	0.860	0.887
Metago	EC2	0.902	0.922
Metago	SP	0.929	0.935
Metago	AT	0.808	0.830
Metago	MM	0.786	0.808
Metago	DM2	0.869	0.885
Metago	HS5	0.938	0.911
Metago	SC4	0.908	0.901
Metago	SC5	0.943	0.965
Metago	SC6	0.924	0.934
PRED_PPI	HS1	0.895	0.948
PRED_PPI	EC1	0.875	0.860
PRED_PPI	DM1	0.763	0.771
PRED_PPI	CE	0.838	0.841
PRED_PPI	SC1	0.672	0.667
SPRINT	HS2	0.881	0.513
TRI_tool	HS3	0.798	0.803
go2ppi-RF	SC2	0.872	0.915
go2ppi-RF	HS4	0.830	0.863
go2ppi-RF	EC2	0.905	0.937
go2ppi-RF	SP	0.885	0.901
go2ppi-RF	AT	0.789	0.875
go2ppi-RF	MM	0.738	0.836
go2ppi-RF	DM2	0.843	0.853
HVSM	HS5	0.784	0.801
HVSM	SC4	0.747	0.777
GIS-MaxEnt	SC5	0.919	0.915
DeepSequencePPI	SC6	0.932	0.942

Buljan, M., Ciuffa, R., van Drogen, A., Vichalkovski, A., Mehnert, M., Rosenberger, G., et al. (2020). Kinase interaction network expands functional and disease roles of human kinases. *Molecular cell* 79, 504–520

Durmuş, S. and Ülgen, K. Ö. (2017). Comparative interactomics for virus–human protein–protein interactions: Dna viruses versus rna viruses. *FEBS open bio* 7, 96–107

Fan, J., Upadhye, S., and Worster, A. (2006). Understanding receiver operating characteristic (roc) curves. *Canadian Journal of Emergency Medicine* 8, 19–20

Gokhale, R. S. and Khosla, C. (2000). Role of linkers in communication between protein modules. *Current opinion in chemical biology* 4, 22–27

Gonzalez, M. W. and Kann, M. G. (2012). Chapter 4: Protein interactions and disease. *PLoS computational biology* 8, e1002819

Jain, S. and Bader, G. D. (2010). An improved method for scoring protein-protein interactions using semantic similarity within the gene ontology. *BMC bioinformatics* 11, 562

Jiang, J. J. and Conrath, D. W. (1997). Semantic similarity based on corpus statistics and lexical taxonomy. *arXiv preprint cmp-lg/9709008*

Koschützki, D. and Schreiber, F. (2008). Centrality analysis methods for biological networks and their application to gene regulatory networks. *Gene regulation and systems biology* 2, GRSB–S702

Li, Z., Ivanov, A. A., Su, R., Gonzalez-Pecchi, V., Qi, Q., Liu, S., et al. (2017). The oncoppi network of cancer-focused protein–protein interactions to inform biological insights and therapeutic strategies. *Nature communications* 8, 1–14

Lin, D. et al. (1998). An information-theoretic definition of similarity. In *Icml* (Citeseer), vol. 98, 296–304

Pekar, V. and Staab, S. (2002). Taxonomy learning-factoring the structure of a taxonomy into a semantic classification decision. In *COLING 2002: The 19th International Conference on Computational Linguistics*

Pesquita, C. (2017). Semantic similarity in the gene ontology. In *The Gene Ontology Handbook* (Humana Press, New York, NY). 161–173

Resnik, P. (1999). Semantic similarity in a taxonomy: An information-based measure and its application to problems of ambiguity in natural language. *Journal of artificial intelligence research* 11, 95–130

Schoenrock, A., Samanfar, B., Pitre, S., Hooshyar, M., Jin, K., Phillips, C. A., et al. (2014). Efficient prediction of human protein-protein interactions at a global scale. *BMC bioinformatics* 15, 383

Schwikowski, B., Uetz, P., and Fields, S. (2000). A network of protein–protein interactions in yeast. *Nature biotechnology* 18, 1257–1261

Von Mering, C., Krause, R., Snel, B., Cornell, M., Oliver, S. G., Fields, S., et al. (2002). Comparative assessment of large-scale data sets of protein–protein interactions. *Nature* 417, 399–403

Wang, J. Z., Du, Z., Payattakool, R., Yu, P. S., and Chen, C.-F. (2007). A new method to measure the semantic similarity of go terms. *Bioinformatics* 23, 1274–1281

Wang, X. and Jin, Y. (2017). Predicted networks of protein-protein interactions in stegodyphus mimosarum by cross-species comparisons. *BMC genomics* 18, 716

Yeger-Lotem, E. and Sharan, R. (2015). Human protein interaction networks across tissues and diseases. *Frontiers in genetics* 6, 257

Yu, G., Li, F., Qin, Y., Bo, X., Wu, Y., and Wang, S. (2010). Gosemsim: an r package for measuring semantic similarity among go terms and gene products. *Bioinformatics* 26, 976–978

Yu, H., Kim, P. M., Sprecher, E., Trifonov, V., and Gerstein, M. (2007). The importance of bottlenecks in protein networks: correlation with gene essentiality and expression dynamics. *PLoS computational biology* 3
